# The RCCM 2009 Survey: Mapping Doctoral and Postdoctoral CAM Research in the United Kingdom

**DOI:** 10.1093/ecam/nep184

**Published:** 2011-06-07

**Authors:** Nicola Robinson, George Lewith

**Affiliations:** ^1^Centre for Complementary Healthcare and Integrated Medicine (CCHIM), Faculty of Health and Human Sciences, Thames Valley University, Paragon House, Boston Manor Road, Brentford, Middlesex TW8 9GA, UK; ^2^Primary Care, University of Southampton Medical School, UK

## Abstract

Complementary and Alternative Medicine (CAM) is widely available in the UK and used frequently by the public, but there is little high quality research to sustain its continued use and potential integration into the NHS. There is, therefore, a need to develop rigorous research in this area. One essential way forward is to train and develop more CAM researchers so that we can enhance academic capacity and provide the evidence upon which to base strategic healthcare decisions. This UK survey identified 80 research active postgraduates registered for MPhils/PhDs in 21 universities and were either current students or had completed their postgraduate degree during the recent UK Research Assessment Exercise (RAE) 2001–2008. The single largest postgraduate degree funder was the university where the students registered (26/80). Thirty-two projects involved randomized controlled trials and 33 used qualitative research methods. The UK RAE also indicates a significant growth of postdoctoral and tenured research activity over this period (in 2001 there were three full time equivalents; in 2008 there were 15.5) with a considerable improvement in research quality. This mapping exercise suggests that considerable effort is currently being invested in developing UK CAM research capacity and thus inform decision making in this area. However, in comparative international terms UK funding is very limited. As in the USA and Australia, a centralized and strategic approach by the National Institute of Health Research to this currently uncoordinated and underfunded activity may benefit CAM research in the UK.

## 1. Introduction

The importance of investigating Complementary and Alternative Medicine (CAM) to provide evidence about its widespread use and effectiveness continues to be controversial in the UK [1]. This is against the background of some opposition to CAM within the UK and the limited funds available to sustain CAM research [2, 3]. A recently published editorial has urged “CAM practitioners to get involved in scientific investigation of their own subject—and that research was too important to be left to the scientists" [4]. The importance of engaging CAM practitioners in the research process was first identified in the House of Lords report [5] and subsequently developed through the CAM doctoral and postdoctoral schemes sponsored by the Department of Health National Centre for coordinating Research Capacity. Nine doctoral and nine postdoctoral fellowships were awarded during 2003 and 2004 to individuals at UK universities. Some of the postdoctoral fellows were from CAM therapy backgrounds, but all of the PhD candidates were CAM therapists [6]. In response to this clear strategic need, professional bodies governing Osteopaths, Chiropractors, Acupuncturists and Chinese Herbal Medicine practitioners responded by providing research funding for databases and supported various practitioner and postgraduate research initiatives [7–9]. CAM STrategy Research ANd Development (CAMSTRAND) was formed in March 2007 to facilitate networking among CAM research and teaching groups in UK universities. There are currently 42 higher education institutions providing undergraduate courses in CAM for 2009 UCAS entry, and many of these universities also support CAM MPhil and PhD students in their research endeavours.

Given the urgent need for evidence in this area it was decided at the CAMSTRAND meeting in York (April 2008) that, in conjunction with Research Council for Complementary Medicine (RCCM), a mapping exercise should take place to quantify the extent of CAM postgraduate research in the UK and the extent to which CAM research was represented in the most recent Research Assessment Exercise (RAE) for the period 2001–08. The RAE is designed to evaluate the scientific quality of particular research groups in UK universities in accordance with their international rank, but with respect to the research area or “unit of assessment" in which they operate (e.g., primary care, nursing and health psychology). There are currently five specific graded categories for the RAE:

4*: Quality that is world-leading in terms of originality, significance and rigor
3*: Quality that is internationally excellent in terms of originality, significance and rigor but nonetheless falls short of the highest standards of excellence
2*: Quality that is recognized internationally in terms of originality, significance and rigor
1*: Quality that is recognized nationally in terms of originality, significance and rigor
Unclassified: Quality that falls below the standard of nationally recognized work, or work that does not meet the published definition of research for the purposes of this assessment

The RAE score is effectively a summary of the quality of each group's research activity and includes all research funding over the RAE period along with its source, the number of postgraduate degrees and successful research fellowships underway and completed, as well as four key scientific papers for each researcher submitted into the exercise. This is, then, evaluated by an independent panel, and the scores are discussed and refined before the final outcome is published.

From the postgraduate perspective we were interested in identifying research with a CAM focus, which may have been located in a range of university departments such as medicine, psychology, biochemistry, phytochemistry, nutrition, anthropology and social science. Identifying the range and extent of postgraduate research may help in providing and facilitating future research opportunities and collaborations as well as allowing us to understand one element of the breadth and scale of current UK CAM research. The recent RAE provides us with an overview of the quality and location of ongoing CAM research with respect to other similar scientific areas as well as some idea of how this field is perceived within the academia.

## 2. Methodology

Data concerning postgraduate research for higher degrees was collected between August and November 2008 using an online questionnaire (surveymonkey.com) that took ∼5 minutes to complete. The questionnaire asked respondents to provide details of their department and organization, the total numbers of postgraduate CAM students and their specialist areas. A filter then directed “heads of departments" to a separate web page to provide details on each of their postgraduate students, including thesis title, methodology, the student's background and funding sources. An email containing the link to the questionnaire and background information to the study was sent out in July 2008, with a reminder sent in September 2008. Our express aim was to map postgraduate (MPhil/PhD) CAM research activity in the UK. We allowed the respondents to define CAM within the broad context of the House of Lords [5] and NCCAM definitions.

Data concerning the RAE (released in December 2008) was collected in January 2009 using the existing contact list identified in the initial part of the survey. We asked not only about the 2008 RAE but also about the responses that had occurred during the RAE in 2001, for purposes of comparison.

Participants were identified primarily through CAMSTRAND, and questionnaires were sent via email link to all those within this network. A snowballing process was then initiated to identify as many CAM researchers as possible. A similar approach was used within the Alternative and Complementary Health Research Network (ACHRN) membership, and also through another survey sent to RCCM members asking for information about research active individuals in the UK. All participants, particularly existing postgraduate students, were also asked to forward the email to any other postgraduate student or supervisor who they knew was involved with CAM research. Using a snowballing approach made it likely that individuals would receive more than one notification about the questionnaire. However, we made it clear that it should be completed only once and that the final data should be checked to avoid any duplication.

The new RCCM strategy identified the need to log this postgraduate CAM research; this survey is ongoing, and the outcome will be regularly updated. See http://www.surveymonkey.com/s.aspx?sm=SXw5hmjZsDXUQasjXbHohA_3d_3d

## 3. Results

The initial survey evaluating postgraduate research was sent to 229 individual email addresses. This was augmented further by asking the people responding to the questionnaire to use their own contact lists to identify more research active individuals. Of the 229 emails sent, 19 were undeliverable and 63 individuals responded; 21 of these were as a result of emails being forwarded on in the manner described. Of these 63 responses, 24 did not have postgraduate CAM students working with them. A total of 21 different universities were identified as having postgraduate CAM researchers (Table 1). We are also aware of a number of projects that have not been included at the individuals' request.  Table 2 indicates the specific field of research for each individual student.

We were able to identify 80 postgraduate CAM students based on project supervisors defining their students' projects as having a CAM focus. According to the supervisors who answered the question about their students' background, 36 students were CAM practitioners, 27 had a biomedical backgrounds (medical, nursing, psychology, biomedical sciences and physiotherapy), and the other 17 came from backgrounds such as sociology, history or anthropology.

Most projects and postgraduate degrees were funded directly by the sponsoring university (26), with 17 recording “other" sources of funding, including medical and associated charities. Fourteen were self-funded and 11 were funded by the Department of Health; the remainder were funded by a research council [6], industry [2], a professional organization [1] or scholarships from a foreign government [2], with data missing for one postgraduate student.

A variety of methodological techniques were employed by the postgraduate researchers. Mixed methods approaches were common, involving several different research methodologies within each project. The methods identified below are, therefore, more frequent than the total number of researchers. Thirty-two individuals were involved in randomized clinical trials; 33 projects involved qualitative approaches; 16 were systematic literature reviews; 12 were engaged in laboratory work; 18 were using case control, cohort or observational studies and 23 have employed questionnaires and/or surveys.

Five of the universities who replied had CAM included in their RAE submissions. (York, Southampton, Westminster, Exeter and Plymouth). The CAM research groups at these universities were within a number of units of assessment in conjunction with a variety of other researchers; this included primary care, community-based clinical disciplines, nursing and midwifery, allied health professionals and health services research. A total of 15.5 full time equivalent (FTE) researchers were returned within the five institutions, and those responding estimated that in some instances up to 30% of the total research submitted within their unit of assessment was CAM based. This compares to the three FTEs in the 2001 RAE. The composite research scores involving CAM ranged from 2 to 4* in 2008, indicating that CAM research can be associated with international research excellence within the UK and its further education assessment systems. Overall this appears to be an increase in activity and also apparent quality, when compared with the previous assessment 6 years ago.

## 4. Discussion

This mapping exercise is the first time that anyone has identified the number of UK universities involved in CAM postgraduate research along with their sources of funding, the general field of endeavor and the type (methodology) of the research being undertaken. Our results appear to suggest that UK universities are far more research active in this area than might be surmised from recent publicity [4], and this activity seems to be growing slowly. However, in an international context, and in relation to the use of CAM by the UK population, this research investment is minimal and unlikely to yield substantial volumes of evidence to inform clinical decision making. Hadley et al. [10] make it clear that there is considerable enthusiasm for CAM research in the UK, but we currently have no formal central structures to facilitate this process. It is pertinent to consider the developments in the USA, where the NIH has approached this area with substantial strategic investment and thought [11] as well as the more recently established National Institute for Complementary Medicine in Australia who has also adopted a long-term strategic approach to this field. It is likely that the rapid expansion of rigorous CAM undergraduate courses in the UK has created a far better understanding of the need for critical appraisal within the clinical CAM community and this in turn may have acted to encourage the development research among CAM practitioners.

This survey method and report have limitations. We chose to survey postgraduate studies as a “signpost" for research activity as well as for identifying as much information as possible from the most recent RAE returns, but we did not attempt to exhaustively map all CAM research in the UK. In relation to the questionnaire, we did have the option of linking email addresses to responses using online survey software, thus only permitting one response per address. However, this was not possible given our need to allow forwarding of the link. We have scrutinized the individual responses to avoid duplication, but it is impossible to identify response rates or bias because of the questionnaire methodology employed. We are aware that there may be other postgraduate students who we have yet to capture within our database, as not all universities in the UK responded; so not all CAM researchers may have been identified. As there is no unit of assessment specifically for CAM in the UK RAE it is impossible to usefully identify more specific information about those involved in CAM research as they are always submitted within other research groups. We are also aware that CAM postgraduate projects are found in many diverse departments. Nevertheless, the information obtained from the RAE indicates that there are a number of centers of research excellence within CAM and that this has grown over the 8 years between the RAEs in 2001 and 2008.

There is an obvious public need to develop a clear strategic approach to CAM research in the UK academic environment, as well as substantial inventiveness and enthusiasm for doing so within the CAM research and practitioner community. We have demonstrated that some effort is being invested in understanding the evidence base for CAM in the UK, but we have not comprehensively mapped the breadth of research being carried out by postdoctoral and tenured researchers, and this will require further study. Furthermore, the pattern of research in this field is inconsistent with the policy suggested by the House of Lords report [5] and appears to be haphazard and opportunistic. We suggest that bodies such as the National Institutes for Health Research should capitalize on this apparent intellectual asset and the existing enthusiasm of the CAM research community by developing the very limited level of current research investment. This would require a thoughtful strategic approach and represent a considered response to the current uninformed debate about CAM in the UK. It could be achieved with the collaboration of conventional medical researchers and would undoubtedly be of benefit to the 10–15% of UK population that use CAM each year [12].

## Funding

Funding for the project was obtained from the Research Council for Complementary Medicine and The Southampton Complementary Medical Research Trust which are both charitable trusts.

## Figures and Tables

**Table 1:** Universities identified as having postgraduate CAM research.

University	Completed CAM postgraduate students	Current CAM postgraduate students
Bournemouth	0	1
Brighton	0	1
Canterbury Christ Church	0	1
Kings College London	0	1
Leeds	0	4
Leeds Metropolitan	0	2
Manchester	1	3
Middlesex University	3	3
Napier University	0	1
Northampton	0	2
Peninsular Medical School (data incomplete)	0	1
Portsmouth	0	1
Plymouth	0	6
Queen Margaret	1	3
Salford	0	1
Sheffield	0	2
Southampton	4	8
Thames Valley	2	8
Ulster	4	3
Westminster	1	6
York	0	6

Total	16	64

**Table 2:** Therapy/field of research.

Acupuncture	21
Mechanisms of action/pharmacology	9
Mind/body therapy	8
CAM general (mainly CAM use for a specific condition)	7
Homeopathy	6
Herbal (including Chinese herbal)	6
Traditional/cultural medicine	4
Practitioner/therapeutic relationship	4
Reflexology	3
Nutrition	3
Osteopathy	2
Chiropractic	2
Kinesiology	1
Missing data	4

Total	80

## References

[1] Adams J (2008). Utilising and promoting public health and health services research in complementary and alternative medicine: the founding of NORPHCAM. *Complementary Therapies in Medicine*.

[2] Lewith GT (2007). Funding for CAM. *British Medical Journal*.

[3] Garrow JS (2007). How much of orthodox medicine is evidence based?. *British Medical Journal*.

[4] Ernst E (2008). Research is too important to be left to the scientists. *Focus on Alternative and Complementary Therapies*.

[5] House of Lords Select Committee on Science and Technology Sixth Report, Session 1999–2000.

[6] Wye L (2008). Dear department of health. *Journal of Holistic Healthcare*.

[7] Robinson N (February 2005). How the BAcC is offering support funding for acupuncture research. *British Acupuncture Council - News*.

[8] Robinson N (June 2005). Research news: building research capacity for the profession. *British Acupuncture Council - News*.

[9] Robinson N, Bovey M (2007). Developing UK acupuncture practitioners’ research capacity. *The Journal of Alternative and Complementary Medicine*.

[10] Hadley J, Hassan I, Khan KS (2008). Knowledge and beliefs concerning evidence-based practice amongst complementary and alternative medicine health care practitioners and allied health care professionals: a questionnaire survey. *BMC Complementary and Alternative Medicine*.

[11] Khalsa PS, Pearson NJ (2007). Financial support for research training and career development in complementary and alternative medicine
from the National Institutes of Health. *Journal of Manipulative and Physiological Therapeutics*.

[12] Thomas KJ, Nicholl JP, Coleman P (2001). Use and expenditure on complementary medicine in England: a population based survey. *Complementary Therapies in Medicine*.

